# Outcomes of K-Clip Implantation for Functional Tricuspid Regurgitation Accompanied with Persistent Atrial Fibrillation

**DOI:** 10.3390/jcdd12020055

**Published:** 2025-02-03

**Authors:** Da-Wei Lin, Ling-Wei Zou, Jia-Xin Miao, Jia-Ning Fan, Min-Fang Meng, Yi-Ming Qi, Zhi Zhan, Wen-Zhi Pan, Da-Xin Zhou, Xiao-Chun Zhang, Jun-Bo Ge

**Affiliations:** 1 Department of Cardiology, Zhongshan Hospital, Fudan University, Shanghai 200032, China; lindaweiii@163.com (D.-W.L.); m_9oo727@163.com (J.-X.M.); fanjianing1999@163.com (J.-N.F.); 23111320005@m.fudan.edu.cn (M.-F.M.); 21111210034@m.fudan.edu.cn (Y.-M.Q.); zhanzhi1991@163.com (Z.Z.); peden@sina.com (W.-Z.P.); jbge@zs-hospital.sh.cn (J.-B.G.); 2 National Clinical Research Center for Interventional Medicine, Shanghai 200433, China; 3 Department of Vascular Surgery, Zhongshan Hospital, Fudan University, Shanghai 200032, China; lingweizou@163.com; 4 Department of Echocardiography, Zhongshan Hospital, Fudan University, Shanghai 200032, China

**Keywords:** transcatheter annular repair, K-Clip™, functional tricuspid regurgitation, atrial fibrillation

## Abstract

**Background:** Atrial fibrillation (AF) has been identified as a risk factor for functional tricuspid regurgitation (FTR) in the absence of other known etiologies, although limited interventional options are available. K-Clip™, a novel transcatheter tricuspid annuloplasty device, is a clip-based annular plication approach for FTR. To date, no studies have investigated the short-term outcomes of K-Clip™ for patients with severe FTR associated with AF. Therefore, the aim of this study was to explore the feasibility and effectiveness of transcatheter annular repair with K-Clip™ for FTR in patients with persistent AF. **Methods:** Patients with FTR and persistent AF who underwent transcatheter annular repair with K-Clip™ at nine centers in China during the inclusion period were included (This study derived from Confirmatory Clinical Study of Treating Tricuspid Regurgitation With K-Clip™ Transcatheter Annuloplasty System [TriStar study}). Baseline data, imaging results, and follow-up data were collected. **Results:** All 52 patients (23 men, 74.02 ± 7.03 years) received successful intervention, and the mean operation time and radian exposure were 2.64 ± 1.09 h and 133.33 ± 743.06 mGy, respectively. No death cases and a low major adverse event occurrence rate were reported in 30 days. A significant decrease in FTR was documented, and TR remained severe in only two patients (3.8%). The regurgitation volume decreased significantly, accompanied by a notable reduction in the effective regurgitation orifice area and tricuspid annulus diameter, which subsequently led to the reversal of right heart remodeling. Furthermore, a decrease in pulmonary artery systolic pressure and an increase in cardiac output were documented. **Conclusions:** Transcatheter annular repair with K-Clip™ showed favorable short-term prognosis and significant improvement in FTR in patients with severe FTR associated with persistent AF. K-Clip™ could be a novel option for that group of patients.

## 1. Introduction

Tricuspid regurgitation (TR) is often overlooked as a secondary pathological consequence of left-sided heart or pulmonary vascular disease despite its prevalence ranking second only to mitral regurgitation and exceeding that of aortic valve disease. Recent research has drawn increased attention to TR, revealing its association with high mortality rates and unfavorable prognostic outcomes [1,2,3,4].

TR encompasses both primary and secondary forms. Primary TR arises from tricuspid valve (TV) abnormalities. Secondary tricuspid regurgitation (functional TR, FTR), which accounts for approximately 90% of cases, primarily stems from annular dilatation and leaflet embolism due to remodeling of the right ventricle or right atrium caused by left-sided heart disease, atrial fibrillation (AF), or pulmonary disease [1,5,6]. Moreover, it is estimated that a considerable proportion of patients, ranging from 5% to 10%, exhibit severe FTR, a condition commonly linked to device leads and AF [5,7]. Furthermore, patients with persistent AF exhibit greater susceptibility to TR than those with paroxysmal AF [8,9,10], and AF-associated FTR may involve right atrial and tricuspid annular enlargement [11,12].

The 2021 ESC/EACTS guidelines for the management of valvular heart disease classify TV surgery as a class I recommendation for patients with severe isolated primary TR who are symptomatic or have right ventricular dysfunction [13]. However, the perioperative mortality rate associated with isolated TV surgery is considerably high, and no notable disparity is found in survival rates when compared with pharmacological therapy [14,15,16]. Transcatheter annular repair is a viable alternative for repairing the tricuspid annulus owing to its notable benefits, including reduced trauma and expedited recovery [17]. However, the efficacy of transcatheter annular repair in managing severe FTR in patients with AF remains unclear.

K-Clip™ (Huihe Company, Shanghai, China) is an innovative device for transcatheter tricuspid annuloplasty, utilizing a clip-based annular plication technique administered through transjugular venous access, emulating Kay’s annuloplasty. This device efficiently reduces the dimensions of the tricuspid annulus without the need to traverse the valve or puncture the leaflets, thereby mitigating potential damage to the valve [18]. The safety and efficacy of K-Clip™ have been validated in animals by Pan et al. and in humans by Zhang et al. [19,20]. However, there are no research findings on the short-term outcomes of K-Clip™ for patients with severe FTR associated with AF. Consequently, we utilized the K-Clip™ device for the treatment of patients with severe FTR and persistent AF and aimed to assess its short-term feasibility and effectiveness.

## 2. Material and Methods

### 2.1. Study Design and Population

This was a retrospective study that included 52 patients with symptomatic severe FTR and persistent AF who were treated with the K-Clip™ annuloplasty system between March 2021 and October 2022. This study was derived from Confirmatory Clinical Study of Treating Tricuspid Regurgitation With K-Clip™ Transcatheter Annuloplasty System ([TriStar]; NCT05173233) study, whcih was conducted at nine centers in China. They were Zhongshan Hospital, Fudan University, Shanghai, China, The First Affiliated Hospital, Zhejiang University, Hangzhou, China, The Second Affiliated Hospital, Zhejiang University, Hangzhou, China, Beijing Anzhen Hospital, Capital Medical University, Beijing, Anhui Provincial Hospital, Hefei, China, Fuwai Hospital, China Academy of Medical Sciences, Guangdong province People’s Hospital, Guangdong, China, The First Affiliated Hospital, Soochow University, Soochow, China. All patients had equal or greater than severe TR (≥4+) and New York Heart Association (NYHA) cardiac function class ≥ II under pharmacological therapy. Furthermore, all patients were diagnosed with persistent AF. The severity of tricuspid regurgitation was assigned a grade of 1 (trace or mild), 2 (moderate), 3 (severe), 4 (massive), or 5 (torrential). Those patients were at great surgical risk as determined by the local heart team, which consisted of board-certified specialists in cardiac surgery, interventional cardiology, echocardiology, and heart failure. Specifically, patients with tricuspid (organic) valve pathology, a pulmonary hypertension (estimated pulmonary artery systolic pressure > 60 mm Hg), right ventricular pacemaker or implantable defibrillator leads, a history of cardiac transplantation, mitral valve prolapse, left ventricular systolic insufficiency (LVEF < 40%), congenital heart disease, rheumatic heart disease, and Ebstein’s malformation were excluded. The baseline information of patients was documented. All individuals in the study provided written informed consent, and the research was approved by the Institutional Review Board of Zhongshan Hospital, Fudan University, Shanghai, China (Number 2022-042).

### 2.2. Echocardiographic and Multi-Detector Computed Tomography (CT) Assessment

FTR was assigned a grade of 1 (mild), 2 (moderate), 3 (severe), 4 (massive), and 5 (torrential), as assessed using standard two-dimensional echocardiography [20,21]. Pre-procedural transthoracic echocardiography, transesophageal echocardiography (TEE), and cardiac CT were conducted separately to evaluate the anatomical suitability for implantation of the K-Clip™ device. All echocardiographic images were analyzed in an independent laboratory.

The target point for anchoring was set by the mid-posterior annulus and moved forward to the anteroposterior commissure when the TR jet derived from the anterolateral annulus was dominant. A cardiac CT scan was utilized for sizing the tricuspid annulus to aid in the selection of implant length and planning of the procedure, thereby reducing the risk of injury to the right coronary artery (RCA) and facilitating the generation of the intended views. The device size was primarily determined by the length of the tricuspid annulus, as measured using cardiac CT, during the maximal diastolic opening of the tricuspid valve.

### 2.3. Investigational Device

Tricuspid transcatheter edge-to-edge valve repair was performed using the K-Clip™ transcatheter tricuspid annuloplasty device, which was the “clamp-annular-screw sandwich structure” formed by two clamp arms and one anchor screw. It is administered via transjugular venous access using an external guide (18F) and a maneuverable inner delivery catheter (15F). The current K-Clip™ device can be used with four different clip arm lengths (12, 14, 16, 18 mm), corresponding to a maximum reduction in annual lengths ranging from 24 mm to 36 mm.

### 2.4. Procedural Technique

**Figure 1:**
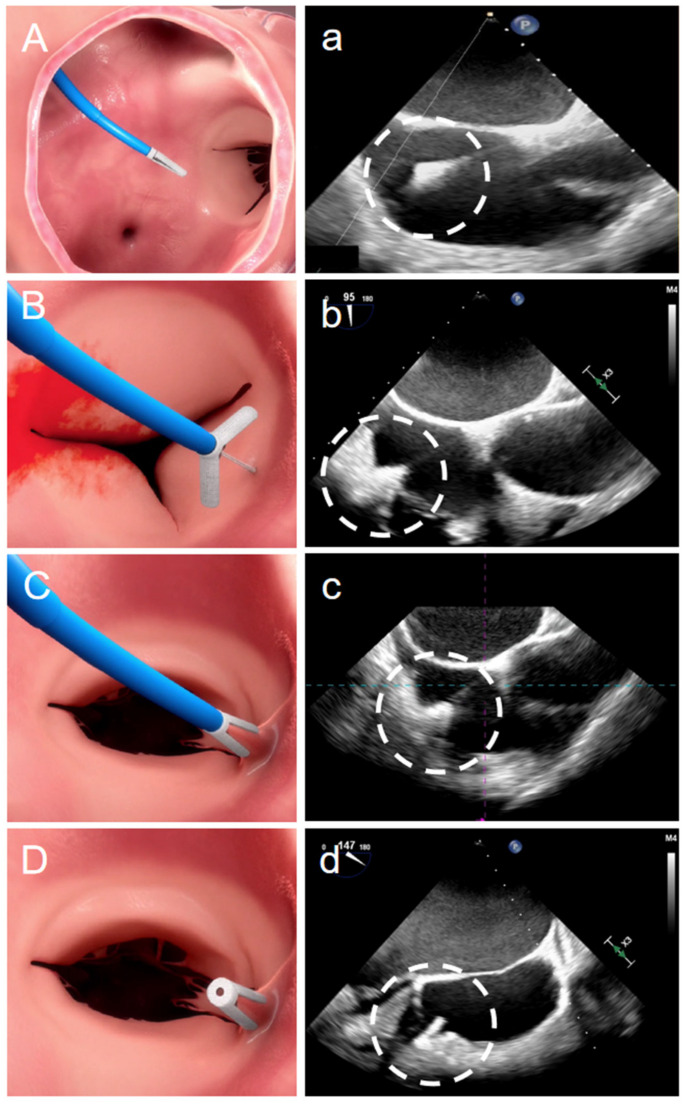
Procedural imaging. (**A**) The K-clip system was introduced percutaneously via the right jugular vein; (**B**) the anchor within the clip was affixed to the mid-posterior annulus; (**C**) the annular tissue was drawn into the clip, and subsequently, the clip closed; and (**D**) the clip was released.

The femoral vein was used as the access point for surgery, with patients undergoing general anesthesia guided by fluoroscopy and TEE. TEE has been used for guidance during the procedure of TR intervention therapies [22,23]. The catheter was guided to the target site in the tricuspid annulus following right coronary angiography, and the anchor located within the clip arm was subsequently inserted into the annulus, at a regulated depth of 4 mm, with echocardiographic guidance from the X-plane. The procedure of transcatheter annular repair with K-Clip™ under TEE was as previously reported and is shown in Figure 1 [24]. Under TEE, the clip arms were opened and positioned tangentially to the tricuspid annulus. The adjacent annulus tissue was drawn into the clip by retracting the anchor. The tooth clip arms were then closed to accomplish sandwiching of the annulus. After RCA coronary angiography and echocardiographic confirmation of TR reduction, the final detachment was performed. Heparin was administered intravenously to attain an active clotting time ranging from 300 to 600 s. After discharge, it was advised that oral anticoagulant therapy be initiated for patients.

### 2.5. Study Endpoints and Follow-Up

Clinical success was defined as procedural success without any major adverse events (MAEs) within a 30-day period. In the present study, MAEs encompassed a range of outcomes including all-cause mortality, cardiovascular mortality, rehospitalization, conversion to surgery, additional minimally invasive interventions, puncture site injury, bleeding events, major vascular complications, worsening of renal insufficiency, stroke, left-sided heart failure, anemia, pericardial effusion, hyperkalemia, myocardial infarction, and a new bundle branch block. The New York Heart Association (NYHA) functional class, 6-min-walk distance (6 MWD), and Kansas City Cardiomyopathy Questionnaire (KCCQ) scores were used as the principal metrics for assessing prognosis (life quality outcome).

Furthermore, the FTR grade and various echocardiographic parameters, such as anatomic and hemodynamic alterations of the right ventricle and tricuspid valve, were acquired at the 30-day follow-up and assessed in an echo core laboratory.

### 2.6. Statistical Analysis

Continuous variables were presented as mean ± standard deviation, and categorical variables were expressed as proportions (%). To compare the baseline and post-procedure follow-up measurements, continuous variables were analyzed using the paired Student’s *t*-test, whereas categorical variables were compared using the Wilcoxon signed-rank test. A two-sided *p*-value of <0.05 was deemed statistically significant. All data analyses were performed using STATA software 15.1 (Stata Corp LP, College Station, TX, USA).

## 3. Results

### 3.1. General Characteristics of Patients

**Figure 2:**
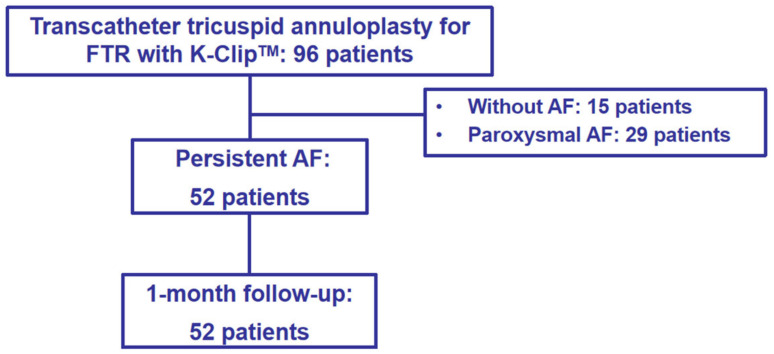
Flowchart of the study.

The baseline characteristics of all enrolled patients are shown in Table 1. A total of 96 patents underwent transcatheter tricuspid annuloplasty with K-Clip^TM^, and 15 subjects and 29 subjects were excluded because of the absence of AF and paroxysmal AF, respectively. As shown in Figure 2, 52 patients (23 men and 29 women) were enrolled in this study, with a mean age of 74.02 ± 7.03 years. Comorbidities included hyperlipidemia (n = 7, 13.4%), hypertension (n = 26, 73.33%), diabetes mellitus (n = 8, 15.3%), peripheral vascular disease (n = 4, 7.6%), renal insufficiency (n = 3, 5.7%), NYHA functional class III or IV (n = 31, 59.6%), myocardial infarction (n = 2, 3.8%), and stroke (n = 13, 25%). Furthermore, 1 patient (1.9%) had previously undergone percutaneous coronary intervention. The medications used at the baseline are presented in Table 2. All patients were prescribed nitrates and diuretics, with additional percentages of 19.2%, 40.3%, and 19.2% of patients also prescribed digoxin, beta-blockers, and calcium channel blockers, respectively.

### 3.2. Procedural Results

During the surgical procedure, essential information regarding the patient surgeries was recorded. As shown in Table 3, 24 patients were implanted with more than two clips (46.1%), and the delivery system was retrieved as intended. All patients achieved procedural success (100%) with a mean operation time and radian exposure of 2.64 ± 1.09 h and 133.33 ± 743.06 mGy, respectively. (Table 3)

### 3.3. In-Hospital and 30-Day Complications

The mean duration of hospitalization was 5.16 ± 3.56 days. The in-hospital and 30-day complications are summarized in Table 4. A low MAE occurrence rate was observed in the study population before discharge. One patient reported major vascular bleeding at the transjugular access site; however, no invasive intervention was required. During the 30-day follow-up, one new incidence of stroke (1.9%), one of left-sided heart failure (1.9%), two of anemia (3.8%), three of pericardial effusion (5.7%).

### 3.4. Improvement in Cardiac Function Between the Baseline and 30-Day Follow-Up

**Figure 3:**
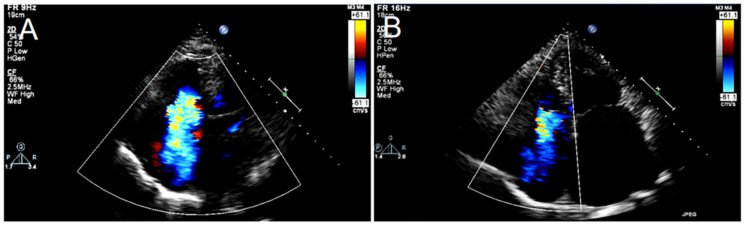
Comparison of TR severity at baseline and 30-day follow-up.

Cardiac ultrasound suggested a significant decrease in TR as the percentage of patients with moderate to severe regurgitation reduced to 26.9%, and severe regurgitation persisted in only two patients (3.8%). As shown in Figure 3 and Table 5, the regurgitation volume decreased from an average of 73.21 ± 53.92 to 29.35 ± 20.78, (*p* < 0.001), combined with a marked reduction in effective regurgitation orifice area (0.99±1.18 vs. 0.34 ± 0.25, *p* < 0.001) and tricuspid annulus diameter (42.48 ± 5.34 vs. 36.17 ± 4.45, *p* < 0.001). Right heart remodeling was reversed, which presented in the right-heart parameters postoperatively with a reduction in right ventricular end-diastolic diameter (left to right, 60.02 ± 11.25 vs. 54.05 ± 11.98, *p* = 0.011; top to bottom, 73.53 ± 13.94 vs. 64.88 ± 14.68, *p* = 0.003) and right ventricular end-diastolic diameter (base, 46.62 ± 7.14 vs. 41.43 ± 6.83, *p* < 0.001). Furthermore, the inferior vena cava width also decreased from 22.93 ± 5.25 to 18.64 ± 6.39 (*p* < 0.001). The PASP reduced from 42.60 ± 7.33 to 35.96 ± 6.90 (*p* < 0.001), whereas CO increased from 4.59 ± 1.65 to 5.53 ± 2.13 (*p* = 0.013).

**Figure 4:**
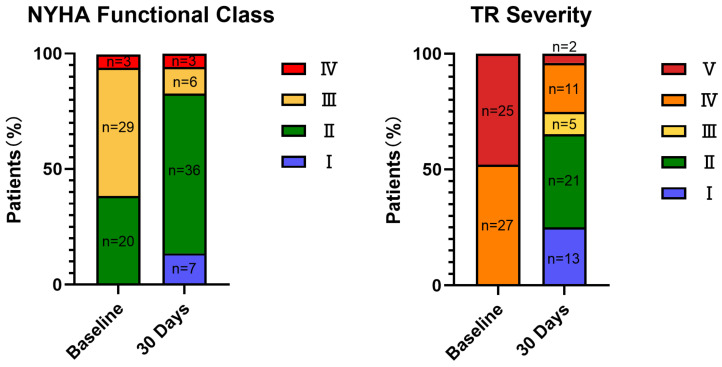
Paired improvements in NYHA functional class and TR severity in 30 days (all *p* < 0.001).

**Figure 5:**
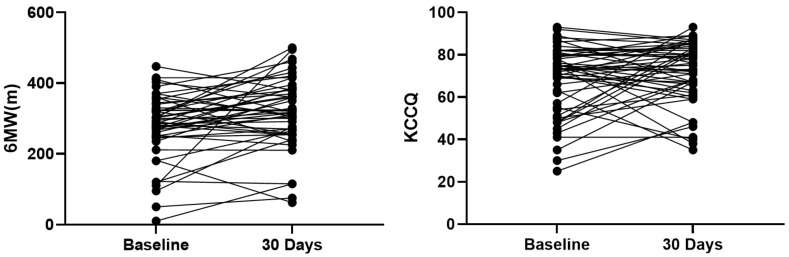
Improvements in KCCQ scores and 6WMD in 30 days (all *p* < 0.001).

For cardiac function assessment, significant improvement in NYHA functional class was observed at the 30-day follow-up (*p* < 0.001) (Figure 4). The prevalence of NYHA functional class ≥ III was significantly reduced (*p* < 0.001). Additionally, the quality of life, according to the KCCQ score, improved significantly (*p* < 0.001) (Figure 4). Despite the lack of significant difference, a favorable trend towards improved exercise capacity based on the 6MWD at 30 days was also observed (Figure 5).

## 4. Discussion

To the best of our knowledge, this is the first study to investigate the short-term outcomes of the K-Clip™ transcatheter annular repair system for patients with severe FTR associated with persistent AF and without structural heart disease. The present study showed low rates of complications and sustained improvement in functional status at the 30-day follow-up, providing evidence of the safety and efficacy of K-Clip™ for severe FTR associated with persistent AF.

Patients with severe FTR and AF are prone to have annular dilatation. In the present study, the TV annular area was significantly larger than normal. The development of FTR can be attributed to several diseases, including AF, left-sided heart disease, and increased right ventricular afterload, with or without pulmonary hypertension. For FTR associated with AF, the annular dilatation is caused by RA dilatation, which, in turn, accelerates the dilatation of the tricuspid annulus. This promotes the persistence of AF; thus TR begets TR, forming a vicious cycle. Utsunomiya et al. used three-dimensional echocardiography to assess FTR and demonstrated that, in patients with chronic AF, the TV annulus area, RA dilatation, and tricuspid leaflet tethering area were larger and smaller, respectively, than those in patients with left-sided heart disease [12].

We demonstrated the short-term safety and efficacy of transcatheter in patients with severe FTR and persistent AF. Current guidelines recommend TV surgery for patients with at least moderate TR who are undergoing left-sided valve surgery (level of evidence I) and for symptomatic patients with severe isolated primary and secondary TR (level of evidence IIa and IIb, respectively) [7]. Furthermore, many studies have reported transcatheter TR treatment in patients after left-sided valve surgery or dysfunction [25]. However, no study has investigated the results of transcatheter treatment in patients with severe AF-related FTR. Because atrial and ventricular dilations both cause TV annulus dilation, a similar treatment indication may also be applied to AF-related FTR. Furthermore, based on Kay’s annuloplasty, the principle of the K-Clip™ system was to reduce the size of the annulus by a figure 8 suture plication of the posterior leaflet, shortening the distance between anterior and posterior-septal leaflets, and resulting in a functional bicuspid valve.

Our results demonstrated that the K-Clip™ annuloplasty system achieved 100% procedural success in all 52 cases, and a low in-hospital or 30-day MAE occurrence rate was reported thereafter. Several types of tricuspid annuloplasty are available, including suture-based (TriAlign (Mitralign Inc., Bost on, MA, USA) and TriCinch (4Tech Cardio, Galway, Ireland) devices), minimally invasive tricuspid annuloplasty with pledget-assisted sutures, and ring-based annuloplasty. The Cardioband device (Edwards Lifesciences, Irvine, CA, USA) has been widely studied and used in clinical practice. Davidson et al. recently reported that the Cardioband device achieved a success rate of 93% without death in the first 30 days and significantly reduced the septolateral tricuspid annular diameter and TR grade. Furthermore, modifications to the reduced anchors, together with the clip-clasping approach, were embedded in the refined design. Based on that, the K-Clip™ system theoretically avoids repeated annulus penetration and offers a flexible and repositionable implantation order to attain TR reduction.

A significant reduction in FTR was observed after treatment with K-Clip™ in patients with severe FTR and persistent AF, with few complications. The size of the right atrium was markedly decreased. However, it remains unclear whether patients should undergo radiofrequency ablation. In patients with AF and TR, AF may lead to atrial remodeling despite undergoing tricuspid annuloplasty because persistent AF has not yet been controlled. Therefore, research with a prolonged follow-up period is needed to investigate long-term prognosis.

## 5. Limitations

This study has certain limitations. First, our sample size was small; more patients should be included in further studies. Second, the duration of the follow-up was short, and our results need to be confirmed over extended periods. Third, no comparison was made between groups of patients without AF and those with paroxysmal AF. Indeed, we are considering a randomized controlled trial that includes such groups. The above conclusions need to be confirmed with the support of more adequate data.

## 6. Conclusions

Tricuspid annuloplasty with K-Clip™ in patients with severe FTR and persistent AF resulted in a favorable short-term prognosis and significant improvement in TR. Therefore, K-Clip™ could be a novel option for those suffering from severe FTR accompanied with AF.

## Figures and Tables

**Table 1:** Baseline characteristics of patients with atrial fibrillation treated with K-clip.

Patient Characteristics	N = 52
Age, yrs	74.02 ± 7.03
Male (n, %)	23/52 (44.2%)
Hyperlipidemia (n, %)	7/52 (13.4%)
Hypertension (n, %)	26/52 (50.0%)
Diabetes mellitus (n, %)	8/52 (15.3%)
Peripheral vascular disease (n, %)	4/52 (7.6%)
Renal insufficiency (n, %)	3/52 (5.7)
Left bundle branch block (n, %)	0/52 (0%)
Right bundle branch block (n, %)	0/52 (0.0%)
Atrioventricular block (n, %)	0/52 (0.0%)
NYHA functional class III or IV (n, %)	32/52 (61.5%)
Myocardial infarction (n, %)	2/52 (3.8%)
Stroke (n, %)	13/52 (25%)
Previous PCI (n, %)	1/52 (1.9%)
Previous CABG (n, %)	0/52 (0.0%)

Abbreviations: NYHA, New York Cardiac Function; PCI, percutaneous coronary intervention; CABG, coronary artery bypass grafting.

**Table 2:** Drug history of patients with atrial fibrillation treated with K-clip.

Drug History	N = 52
digoxin (n, %)	10/52 (19.2%)
Betaloc (n, %)	21/52 (40.3%)
calcium channel blockers (n, %)	10/52 (19.2%)
nitrates (n, %)	52/52 (100%)
diuretics (n, %)	52/52 (100%)

**Table 3:** Surgery information of patients with atrial fibrillation treated with K-clip.

Surgery Information	N = 52
implantation of clips ≥ 2 (n, %)	24/52 (46.1%)
total duration of surgery (hour)	2.64 ± 1.09
radiant exposure/mGy	1100 (1456.5, 850)
duration of hospitalization (day)	5.16 ± 3.56

**Table 4:** Clinical end points of patients with atrial fibrillation treated with K-clip.

Clinical End Points	In-Hospital	30-Days
	N = 52	N = 52
**Primary endpoints**		
All-cause mortality (n, %)	0 (0.0%)	0 (0.0%)
Cardiovascular mortality (n, %)	0 (0.0%)	0 (0.0%)
**Secondary endpoints**		
Re-hospitalization (n, %)	-	0 (0.0%)
Converted to surgery (n, %)	0 (0.0%)	0 (0.0%)
Additional minimally invasive interventions during (n, %) Hospitalization (n, %)	0 (0.0%)	0 (0.0%)
Puncture site injury (n, %)	0 (0.0%)	0 (0.0%)
Bleeding event (n, %)	0 (0.0%)	1 (1.9%)
Major vascular complication (n, %)	1 (1.9%)	0 (0.0%)
Worsening of renal insufficiency (n, %)	0 (0.0%)	0 (0.0%)
Stroke (n, %)	0 (0.0%)	0 (0.0%)
Left heart failure (n, %)	0 (0.0%)	1 (1.9%)
Anemic (n, %)	0 (0.0%)	2 (3.8%)
Pericardial effusion (n, %)	0 (0.0%)	3 (5.7%)
Hyperkalemia (n, %)	0 (0.0%)	0 (0.0%)
Hypokalemia (n, %)	0 (0.0%)	0 (0.0%)
Myocardial infarction (n, %)	0 (0.0%)	0 (0.0%)
New brunch block (n, %)	0 (0.0%)	0 (0.0%)
New atrioventricular block	0 (0.0%)	0 (0.0%)

**Table 5:** Comparison between baseline and 30-day imaging information of patients with atrial fibrillation treated with K-clip.

	Baseline (N = 52)	30 Days (N = 52)	*p-*Value
**Echocardiography**			
RAED (left to right) (mm)	60.02 ± 11.25	54.05 ± 11.98	0.011
RAED (top to bottom) (mm)	73.53 ± 13.94	64.88 ± 14.68	0.003
RVED (middle) (mm)	34.00 ± 6.70	31.04 ± 6.12	0.020
RVED (base) (mm)	46.62 ± 7.14	41.43 ± 6.83	<0.001
Tricuspid annulus diameter (anterior to posterior) (mm)	42.48 ± 5.34	36.17 ± 4.45	<0.001
Diameter (septal side) (mm)	42.77 ± 5.47	37.63 ± 5.55	<0.001
Reduced flow path width (mm)	12.40 ± 4.66	6.22 ± 3.66	<0.001
Moderate to severe regurgitation (VC)	52/52 (100%)	14/52(26.9%)	<0.001
Effective regurgitation orifice area (cm^2^)	0.99 ± 1.18	0.34 ± 0.25	<0.001
Moderate to severe regurgitation (PISA)	52/52 (100%)	17/52 (32.7%)	<0.001
Moderate to severe regurgitation (combined PISA and VC)	52/52 (100%)	16/52 (30.8%)	<0.001
Regurgitation Volume (cm^3^)	73.21 ± 53.92	29.35 ± 20.78	<0.001
Mean transvalve pressure (mmHg)	1.10 ± 0.45	1.15 ± 0.50	0.539
TAPSE (mm)	18.05 ± 3.09	18.08 ± 5.31	0.973
FAC (%)	39.48 ± 7.02	39.68 ± 10.66	0.909
Inferior vena cava width (mm)	22.93 ± 5.25	18.64 ± 6.39	<0.001
PASP (mmHg)	42.60 ± 7.33	35.96 ± 6.90	<0.001
LVEF (%)	60.77 ± 7.33	61.46 ± 6.58	0.615
LVEDd (cm^3^)	103.29 ± 30.88	108.48 ± 33.22	0.412
LVEDs (cm^3^)	40.53 ± 14.88	38.67 ± 15.06	0.528
CO (mL/min)	4.59 ± 1.65	5.53 ± 2.13	0.013

Abbreviations: RAED: right atrial end-diastolic diameter; RVED: right ventricular end-diastolic diameter; FAC: fractional area change; PASP: pulmonary artery systolic pressure; LVEF: left ventricular ejection fraction; LVEDd: left ventricular end-diastolic dimension; LVEDs: left ventricular end-systolic dimension; CO: cardiac output.

## Data Availability

The data that support the findings of this study are available from the corresponding author upon reasonable request.
